# Comparative metabolomics of two nettle species unveils distinct high-altitude adaptation mechanisms on the Tibetan Plateau

**DOI:** 10.1186/s12870-025-06666-9

**Published:** 2025-05-15

**Authors:** Li-Juan Deng, Yin-Lei Li, Feng-Ying Wang, Xiang-Qian Sun, Richard I. Milne, Jie Liu, Zeng-Yuan Wu

**Affiliations:** 1 Germplasm of Bank of Wild Species & Yunnan Key Laboratory of Crop Wild Relatives Omics, Kunming Institute of Botany, Chinese Academy of Sciences, Kunming, Yunnan 650201 China; 2 CAS Key Laboratory for Plant Diversity and Biogeography of East Asia, Kunming Institute of Botany, Chinese Academy of Sciences, Kunming, Yunnan 650201 China; 3 National Engineering Laboratory for Resource Developing of Endangered Chinese Crude Drugs in Northwest of China, College of Life Sciences, Shaanxi Normal University, Xi’an, 710062 China; 4 School of Ecology and Environment Science, Yunnan University, Kunming, China; 5 University of Chinese Academy of Sciences, Beijing, China; 6 Institute of Molecular Plant Sciences, School of Biological Sciences, University of Edinburgh, Edinburgh, EH9 3JH UK; 7 Department of Biological Sciences, University of Toronto-Scarborough, Toronto, ON Canada

**Keywords:** Tibetan Plateau, *Urtica hyperborea*, *U. dioica*, High-altitude adaptation, Metabolomics, Environmental stress

## Abstract

**Background:**

The extreme high-altitude conditions of the Tibetan Plateau, characterized by intense solar radiation, low temperatures, and reduced oxygen levels, poses significant challenges to plant survival. Plants inhabiting this region have evolved specialized mechanisms to adapt to high-altitude environments. While most studies have focused on genomic and ecological perspectives, few have explored adaptive mechanisms in a metabolic context. In particular, comparative studies examining similarities and differences in the metabolomes of closely related species are exceedingly rare. As sister species, the nettle species *Urtica hyperborea* and *U. dioica* are distributed above 4000 m above sea level, with a sympatric distribution on the Tibetan Plateau, they provide an ideal system to investigate the aforementioned question.

**Results:**

In this study, we conducted non-targeted metabolic profiling of the leaves from *U. hyperborea* and *U. dioica* collected at three sympatric sites on the Tibetan Plateau. A total of 2906 annotated metabolites were detected. Differential metabolites at Sites 1 (4697 m) and 3 (4465 m) were enriched in pathways for flavonoid, flavone and flavonol, and phenylpropanoid biosynthesis. In contrast, Site 2, located at the highest altitude (5007 m), primarily exhibited enrichment in carbon metabolism pathways. Regarding the altitudinal variation of the same species, common metabolic pathways between the two groups included fructose and mannose metabolism, α-linolenic acid metabolism, and glycerophospholipid metabolism. The metabolic pathways enriched only in*U. hyperborea*included starch and sucrose metabolism, galactose metabolism, and phenylpropanoid biosynthesis. The metabolically enriched pathways specific to*U. dioica*included pantothenate and coenzyme A biosynthesis, as well as glutathione metabolism.

**Conclusions:**

We found that the metabolic differences between the two sympatric species are primarily in carbohydrate and phenylpropanoid contents. The differential metabolites of the same species across different altitudes were enriched mainly in carbon metabolism pathways and lipid metabolism pathways. Thus, our study revealed that the high-altitude adaptation mechanisms of sympatric species are not identical. Moreover, adaptation strategies within the same species were generally consistent across altitudes, exhibiting only slight variations. This study provide novel insights into the adaptive metabolic strategies of *U. hyperborea* and *U. dioica*, contributing to a deeper understanding of the mechanisms underlying plant adaptation to extreme high-altitude conditions.

**Supplementary Information:**

The online version contains supplementary material available at 10.1186/s12870-025-06666-9.

## Introduction

The Tibetan Plateau, recognized as the highest and largest plateau on Earth [1, 2], presents a distinctive set of environmental challenges for plants and other life, including intense solar radiation, low temperatures, reduced levels of oxygen and carbon dioxide, and variable humidity and precipitation patterns. Native plant species have evolved various morphological adaptations to thrive in this harsh environment. For example, species such as *Rheum nobile* and *Saussurea involucrata* [3, 4] have developed bracts that minimize UV penetration, whereas *Eriophyton wallichii* [5] features pubescent leaves that not only retain heat but also mitigate pollen damage caused by high temperatures under intense light. Despite these challenging conditions, the Tibetan Plateau and adjacent mountain regions rank among the most biodiverse regions on the planet [6]. However, from a metabolic perspective, it remains unclear whether closely related plants adopt similar evolutionary strategies or develop unique adaptive mechanisms when confronted with analogous environmental pressures.

*Urtica hyperborea* Jacq. ex Wedd. is a perennial herbaceous plant of the *Urtica* genus, primarily distributed in and around the Tibetan Plateau (i.e., the Third Pole) [2], occurring between 3000 and 5200 m a.s.l [7]. It features lignified, thick underground stems and reaches a height of 15–50 cm, with its entire structure densely armed with stinging hairs [7]. As a sister specie to *U. hyperborean* [8, 9], *U. dioica* L. is a cosmopolitan plant. It is also a perennial herb of the *Urtica* genus, fully covered with stinging hairs, and grows to a height of 40–100 cm [7, 10]. Based on our fieldwork observation, *U. hyperborea* and *U. dioica* often exhibit sympatric distribution patterns on the Tibetan Plateau. Therefore, *U. hyperborea* and *U. dioica* provide an ideal model for investigating the similarities and differences in the mechanisms of plant adaptation to high-altitude environments in closely related sympatric species.

In high-altitude regions, extreme environmental conditions such as low temperatures, drought, and intense radiation pose significant challenge to plant survival. Under low-temperature stress, plants mitigate freezing injury by accumulating soluble sugars and unsaturated fatty acids [11–14]. UV radiation damages chloroplasts and DNA [15], howevwe alpine plants counteract this damage by producing UV-absorbing compounds such as flavonoids and anthocyanin glycosides, which mitigate UV-B damage by scavenging reactive oxygen species [16–18]. Drought stress further exacerbates the survival challenges for plateau plants. Drought-tolerant species such as quinoa and chickpeas accumulate proline, histidine, unsaturated fatty acids, and phospholipids as protective strategies [19, 20]. Similarly,tomatoes enhance drought resistance via the synthesis of phenylamide and the upregulation of antioxidant enzyme activity [21].

In addition to controlled experiments examining the effects of abiotic stress on plants, many studies have also utilized wild-collected materials to investigate plant adaptation mechanisms to high-altitude environments. For instance, the chemical composition of *Dendrobium officinale* varies with altitude, with plants from higher altitudes containning higher levels of polysaccharides and exhibiting elevated expression of amino acids and their derivatives [22]. Similarly, *Fritillariae cirrhosae* from higher altitudes accumulate more steroidal alkaloids than those from lower elevations [23]. In *Draba oreades*, metabolic pathways related to flavonoid biosynthesis were found to be upregulated in high-altitude populations, with flavonoid content increasing along the altitudinal gradient [24]. Likewise, *Codonopsis pilosula* tends to accumulate more triterpenes at higher altitudes, which is associated with the upregulation of key enzyme genes [25]. A study on *Zanthoxylum planispinum* also revealed that plants from high altitudes accumulated greater amounts terpenoids, aldehydes, and esters, whereas those from lower elevations are richer in flavonoids and polyphenols [26].

In the study of high-altitude plants, advanced techniques such as metabolomics, transcriptomics, and genomics are increasingly employed to gain deeper insights into plant adaptive mechanisms. However, comparative studies on the adaptive mechanisms of closely related plants remain particularly scarce and warrant further investigation.

In this study, a non-targeted metabolomics approach was used to analyse metabolic diversity in the leaves of *U. hyperborea* and *U. dioica* collected from three sites at different altitudes on the Tibetan Plateau. By identifying differential metabolites and conducting KEGG pathway enrichment analysis, we aimed to: (1) investigate the variations in metabolites between two species within the same site; (2) explore the metabolic variations in the same species across different elavations. Our findings contribute to a better understanding of the metabolic changes and regulatory mechanisms underlying plant adaptation to high-altitude environments.

## Methods

### Study sites and field sampling

**Fig. 1:**
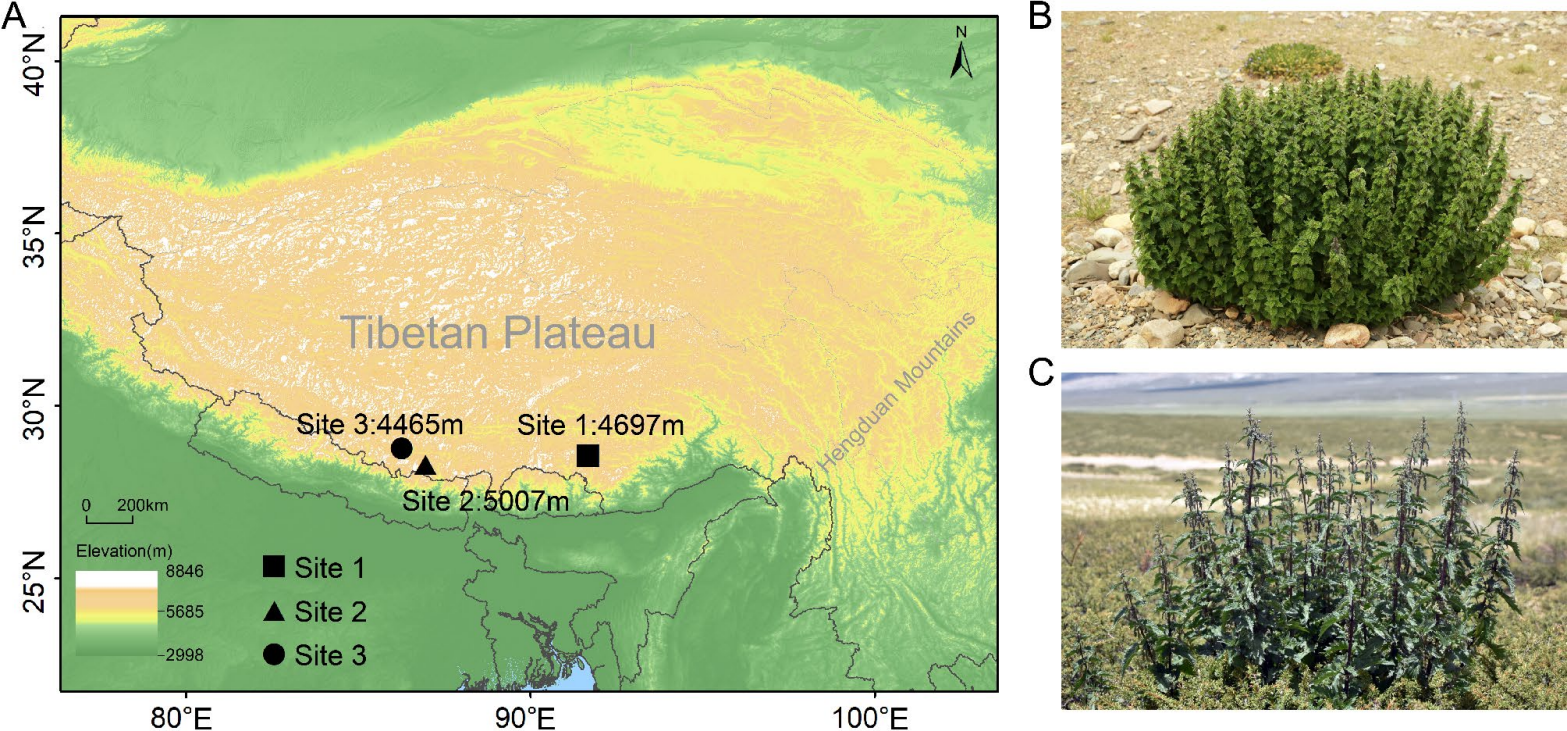
Sampling sites and species photographs. (**A**) Map of the three sampling sites on the Tibetan Plateau, each represented by different symbols. (**B**) Habit and habitat of *U. hyperborea*. (**C**) Habit and habitat of *U. dioica*

In August 2023, leaf samples of *Urtica hyperborea*, and *U. dioica* were collected from three sites where both species co-occur: Comai County, Shannan City, Tibet (Site 1; altitude: 4697 m; coordinates: 91°31’54.97"E, 28°26’42.2"N); Mount Qomolangma, Tingri County, Xigazé City, Tibet (Site 2; altitude: 5007 m; coordinates: 86°49’45.92"E, 28°11’37.09"N); and Nyalam County, Xigazé City, Tibet (Site 3; altitude: 4465 m; coordinates: 86°10’29.96"E, 28°45’2.9"N) (Fig. 1). The specific information for each sample can be found in Table S1.

Samples of *U. hyperborea* from sites 1 to 3 were labeled as 1 H, 2 H, and 3 H, respectively, whereas samples of *U. dioica* were labelled as 1D, 2D, and 3D. At each site, three individuals of each species were collected within a 20 m × 20 m area, resulting in a total of 36 individuals. Following collection, the samples were thoroughly rinsed with distilled water, immediately frozen in liquid nitrogen, and subsequently stored at -80 °C pending for metabolites analysis.

## Metabolite extraction and detection

Approximately 20 mg of liquid nitrogen preserved leaves from each sample were lyophilized and mixed with beads and 1000 µL of extraction solution (MeOH: ACN: water, 2:2:1 v/v) containing deuterated internal standards. The mixture was vortexed for 30 s, homogenized at 35 Hz for 4 min, and sonicated for 5 min in a 4 °C water bath. This process was repeated three times. The samples were then incubated at -40 °C for 1 h to precipitate proteins, followed by centrifugation at 12,000 rpm (RCF = 13,800 × g, *R* = 8.6 cm) for 15 min at 4 °C. The resulting supernatant was transferred to a fresh glass vial for analysis. The quality control samples were prepared by mixing equal aliquots of the supernatants from all the samples.

For the analysis of non-polar metabolites, LC-MS/MS was conducted using a UHPLC system (Thermo Fisher Scientific, Waltham, MA, USA) equipped with a Phenomenex Kinetex C18 column (2.1 mm × 50 mm, 2.6 μm) coupled to an Orbitrap Exploris 120 mass spectrometer (Thermo Fisher Scientific, Waltham, MA, USA). The mobile phase consisted of 5 mmol/L ammonium acetate and 5 mmol/L acetic acid in water (solvent A) and acetonitrile (solvent B). The auto-sampler was maintained at 4 °C, with an injection volume of 2 µL. The Orbitrap Exploris 120 was operated in information-dependent acquisition mode using Xcalibur software, which continuously evaluated the full scan MS spectrum. Electrospray Ionization source conditions were set as follows: sheath gas flow rate at 50 Arb, auxiliary gas flow rate at 15 Arb, capillary temperature at 320 °C, full MS resolution at 60,000, MS/MS resolution at 15,000, collision energy at SNCE 20/30/40, and spray voltage at 3.8 kV (positive) or -3.4 kV (negative).

## Metabolomics data analysis

The raw data were converted to mzXML format via ProteoWizard. The XCMS software [27] is used for peak detection, peak extraction, peak alignment, and integration processing. An In-house MS2 database BiotreeDB (V3.0) was applied to metabolite annotation [28].The detected metabolite signals were classified according to the Metabolomics Standards Initiative [29], and Level 4 metabolites were excluded from further analysis.

The data obtained from the LC-ESI-MS/MS system were normalized via log2 transformation, standardized with z-scores, and subsequently exported to SIMCA software (v16.0.2) [30] for differential metabolite analysis. Quality control (QC) samples, consisting of a mixture of all sample extracts, were included in the analysis queue to monitor method stability. The number of differential metabolites is depicted via a volcano plot constructed with ggplot (v3.3.5) (https://cran.r-project.org/web/packages/ggplot2/index.html), alongside a Venn diagram created via VennDiagram (v1.7.3) [31]. Pathway enrichment analysis of these metabolites was conducted using the Kyoto Encyclopedia of Genes and Genomes (KEGG) pathway database (http://www.kegg.jp/kegg/pathway.html) [32].

### Statistical analysis

OPLS-DA and PLS-DA were performed using SIMCA (v16.0.2). Differential metabolites between groups were identified based on variable importance in projection (VIP) values, *p-*values, and fold change (FC) criteria (VIP > 1, *p* < 0.05, FC ≥ 2 or ≤ 0.5). One-way ANOVA or two-tailed Student’s *t*-tests were conducted to determine significant differences between the two species and among different sites.

## Results

### Untargeted metabolite profiling of *Urtica hyperborea* and *U. dioica*

Using an untargeted metabolomics approach, a total of2,906 high-quality annotated metabolites were identified from 7,436 ion features (Table S2). The total ion chromatograms (TIC) for all samples demonstrated a high degree of overlap, indicating the reliability and consistency of the collected MS data. Analyses of m/z width and retention time width further confirmed that both sample preparation and instrument conditions met the required quality standards (Fig S1, Fig S2).

**Fig. 2:**
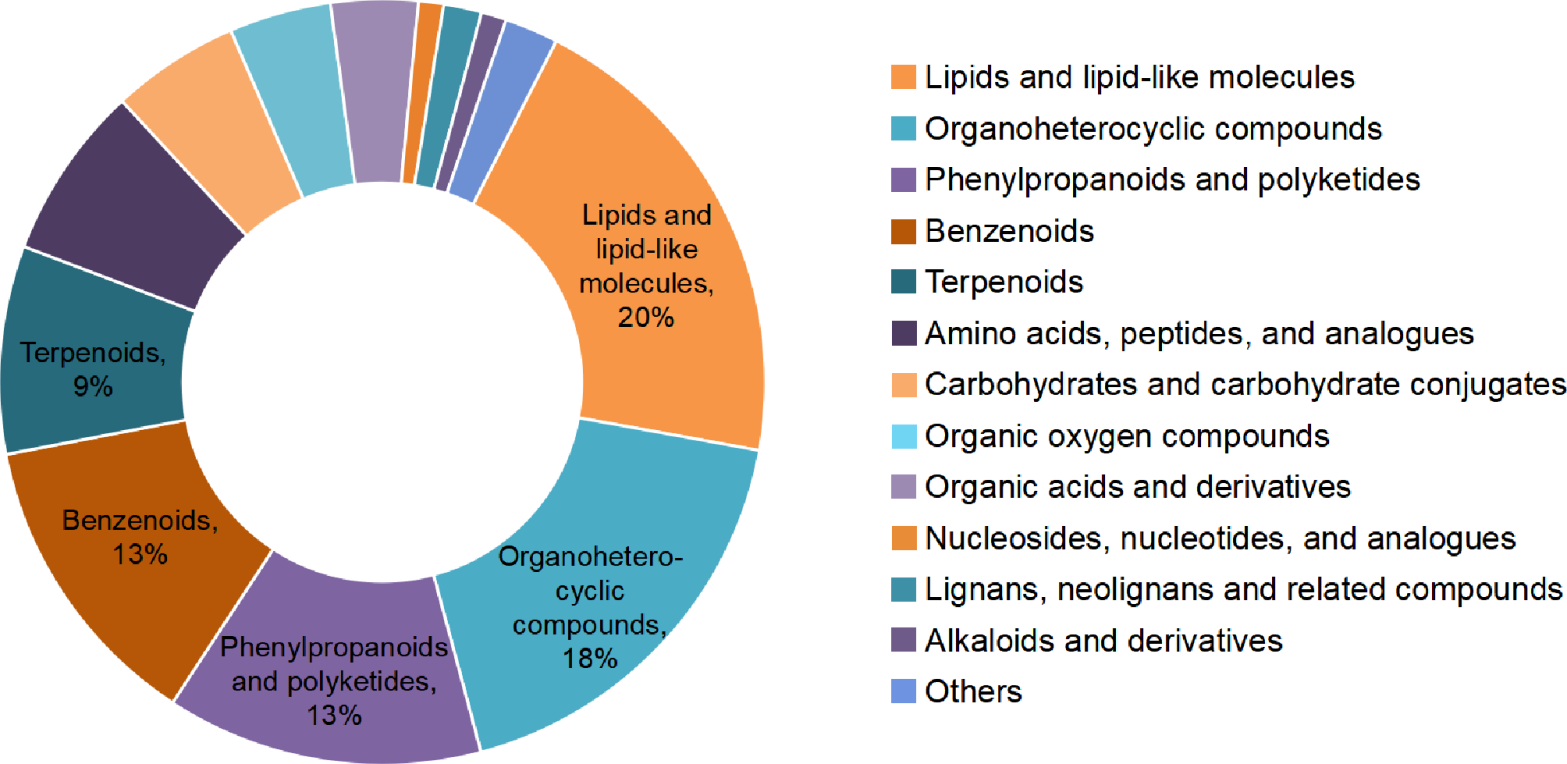
Composition of identified metabolites in *Urtica hyperborea* and *U. dioica*. Thirteen superclasses of metabolites were annotated, with each color in the right-hand legend representing a different superclass. The proportion of the top five superclasses are prominently highlighted

Metabolite annotations revealed a total of 13 metabolic superclasses. The top five superclasses were Lipids and lipid-like molecules (459 metabolites), Organoheterocyclic compounds (406 metabolites), Phenylpropanoids and polyketides (301 metabolites), Benzenoids (288 metabolites), and Terpenoids (198 metabolites) (Fig. 2).

### OPLS-DA analysis of *Urtica hyperborea* versus *U. dioica*

**Fig. 3:**
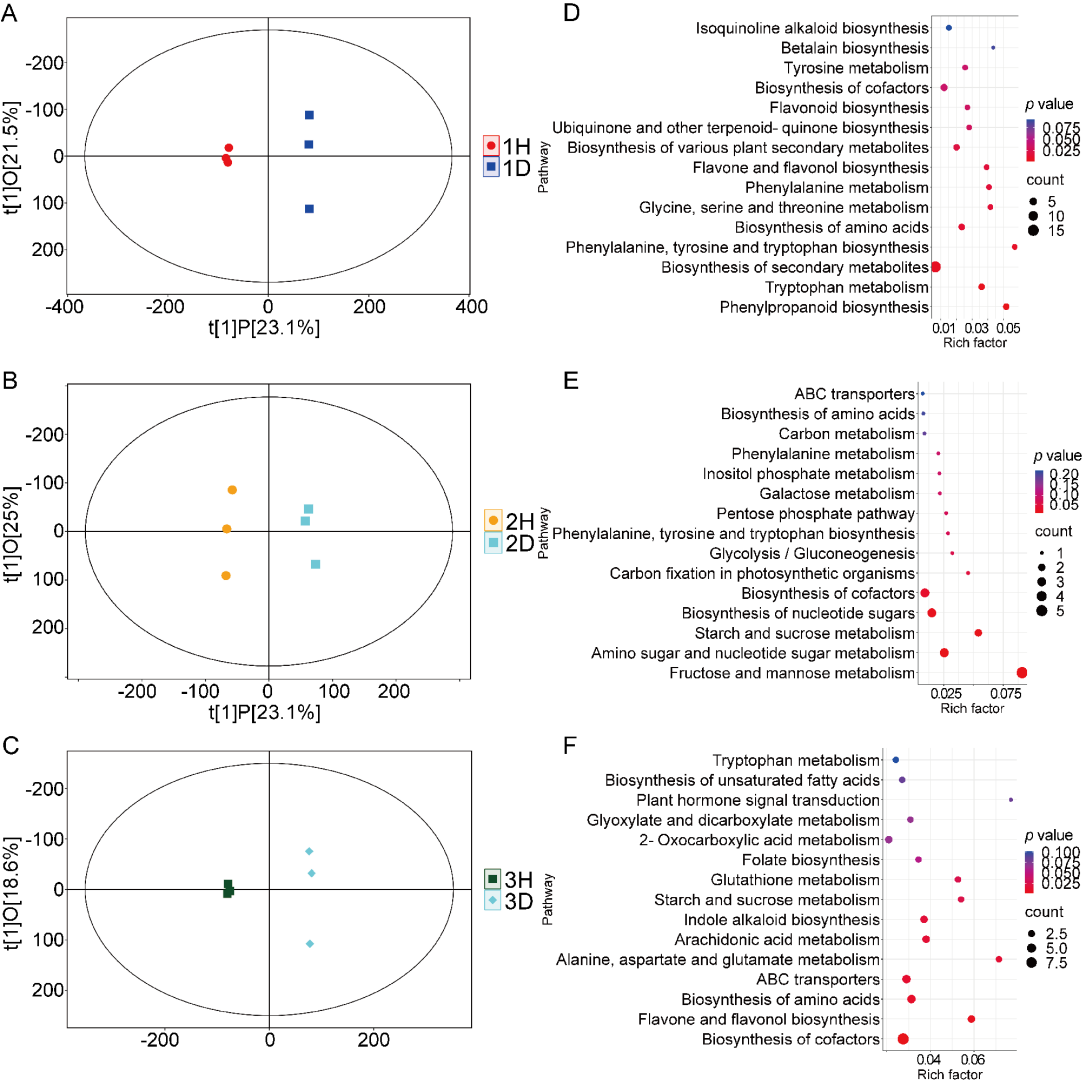
Orthogonal Partial Least Squares Discriminant Analysis (OPLS-DA) and KEGG pathway enrichment analysis of *Urtica hyperborea* (H) and *U. dioica* (**D**) across different sites. (**A**-**C**) OPLS-DA score plots comparing the metabolic profiles of *U. hyperborea* (H) and *U. dioica* (**D**) at three sites: (**A**) Site 1 (1H vs. 1D), (**B**) Site 2 (2H vs. 2D), and (**C**) Site 3 (3H vs. 3D). (**D**-**F**) KEGG pathway enrichment analyses of differentially expressed metabolites between the two species at each site: (**D**) Site 1 (1H vs. 1D), (**E**) Site 2 (2H vs. 2D), and (**F**) Site 3 (3H vs. 3D)

Orthogonal projections to latent structures-discriminant analysis (OPLS-DA) was employed to enhance classification and better interpret intergroup differences [33]. The analysis yielded significant differences between the two species at each of the three sites, in each case with all samples falling within the 95% confidence interval (Hotelling’s *T*-squared ellipse), indicating notable metabolic phenotypic differences (Fig. 3A, B and C).To assess the reliability of the OPLS-DA model and to avoid overfitting, a permutation test was performed, with the results shown in Fig S3. The statistical parameters of the original model were significantly higher than those of the permuted models, indicating strong discriminative power and the absence of overfitting.

### Different metabolites screenings for *Urtica hyperborea* versus *U. dioica*

**Fig. 4:**
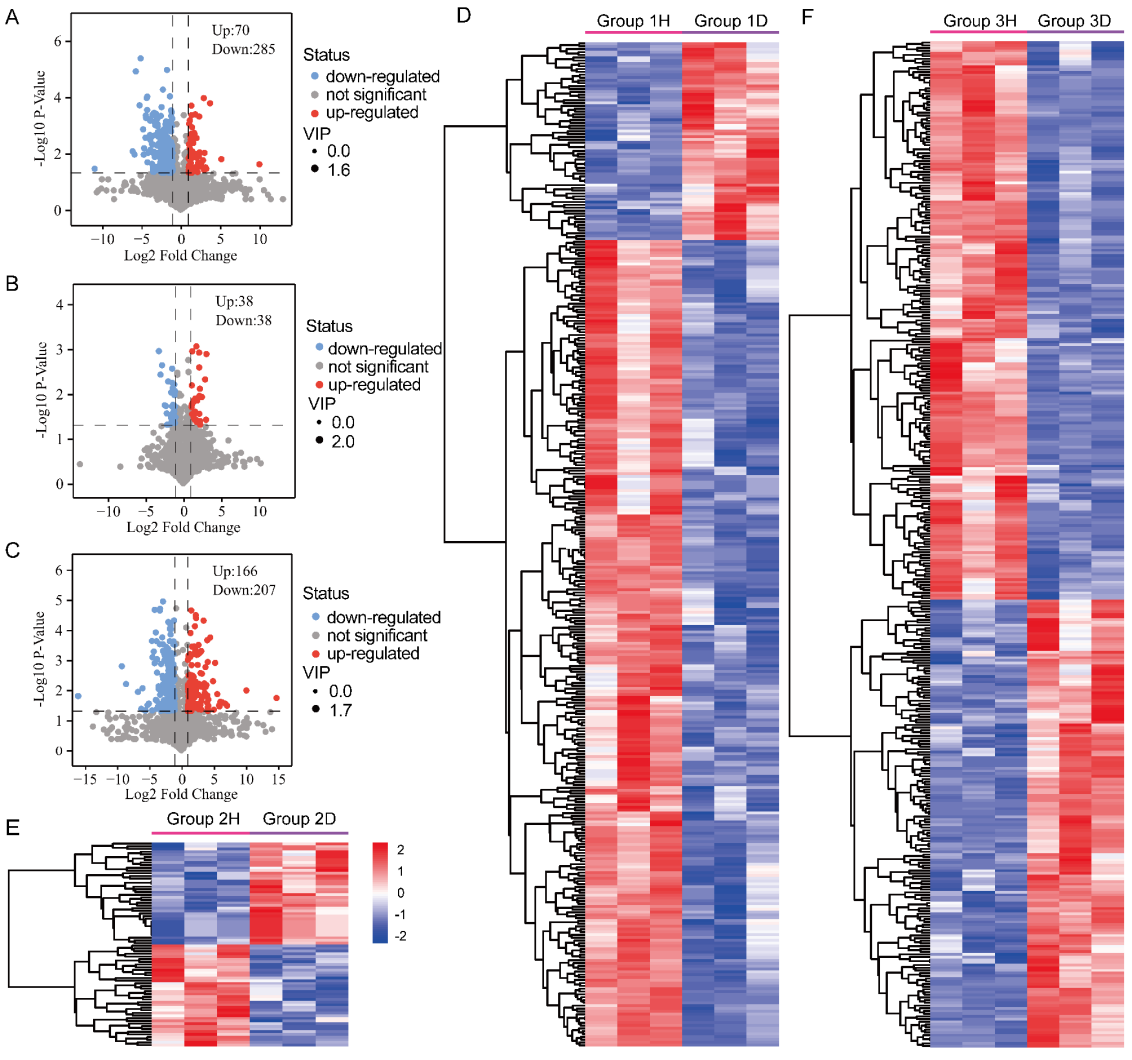
Metabolite Differences Between *Urtica hyperborea* and *U. dioica.* (**A**-**C**) Volcano plots highlighting differentially abundant metabolites (DAMs) between *U. hyperborea* (H) and *U. dioica* (**D**) at three sites: (**A**) Site 1 (1H vs. 1D), (**B**) Site 2 (2H vs. 2D), and (**C**) Site 3 (3H vs. 3D). (**D**-**F**) Heatmaps illustrating the DAMs for each comparison group: (**D**) 1H vs. 1D, (**E**) 2H vs. 2D, and (**F**) 3H vs. 3D

In pairwise comparisons, differential metabolites (DMs) were identified based on criteria of VIP > 1 and FC ≥ 2 or ≤ 0.5. As shown in Fig. 4A, a total of 355 DMs were identified in the comparison between 1 H and 1D(Site 1), including 70 up-regulated and 285 down-regulated metabolites. At Site 2, 76 DMs were identified, with equal counts of up-regulated and down-regulated metabolites (Fig. 4B). At Site 3, 373 DMs were identified, with 166 up-regulated and 207 down-regulated metabolites (Fig. 4C).

To visually illustrate the differences in relative metabolite abundance among groups, a hierarchical clustering heatmap was generated. Heatmap analysis illustrated the relative abundances of DMs among groups 1 H and 1D, 2 H and 2D, and 3 H and 3D. In all three comparisons, the red and blue sections representing the H and D groups, respectively, were clearly separated, indicating significant differences in relative metabolite abundances(Fig. 4D-F).

### KEGG annotation and metabolic pathway analysis of DMs for *Urtica hyperborea* versus *U. dioica*

In biological systems, metabolites do not function in isolation; instead, diverse metabolites interact within complex metabolic networks to regulate life processes [34]. Metabolic pathway enrichment analysis of DMs can provide deeper insights into the regulatory mechanisms underlying biological processes. At Site 1, the metabolic pathways of the DMs are primarily focused on coumarin biosynthesis, tryptophan metabolism, and flavonoid and flavonol biosynthesis (Fig. 3D). At Site 2, the metabolic pathways of the DMs are mainly centered around phenylalanine, tyrosine, and tryptophan biosynthesis, galactose metabolism, starch and sucrose metabolism, fructose and mannose metabolism, and the pentose phosphate pathway (Fig. 3E). At Site 3, the metabolic pathways of the DMs are predominantly involved in glutathione metabolism, indole alkaloid biosynthesis, and flavonoid and flavonol biosynthesis (Fig. 3F).

### DMs screening of the same species at different altitudes

**Fig. 5:**
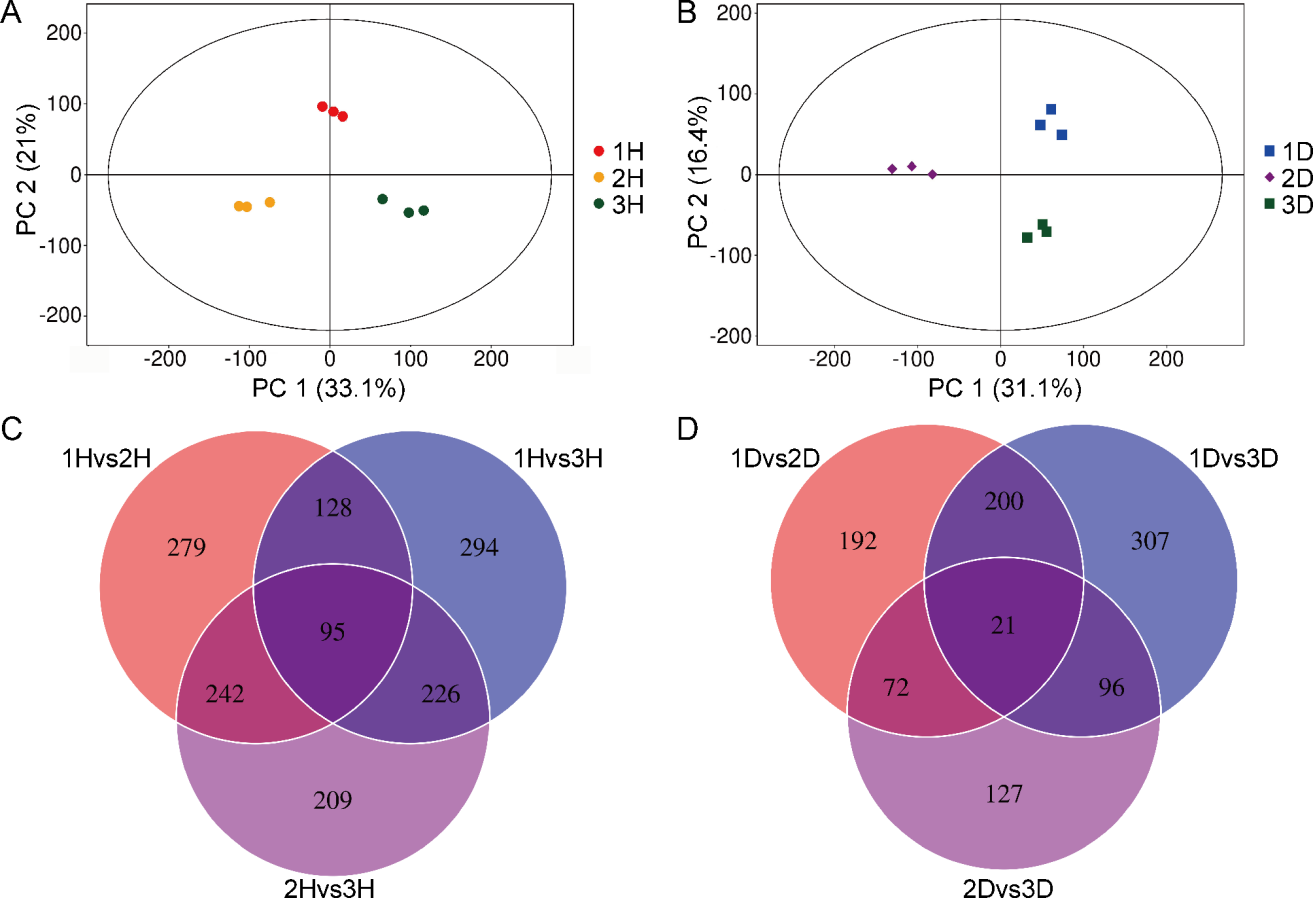
Metabolite differences within the same species across three sites. (**A**-**B**) Partial Least Squares Discriminant Analysis (PLS-DA) score plots comparing metabolite profiles across the three sites: (**A**) *Urtica hyperborea* (1 H vs. 2 H vs. 3 H) and (**B**) *U. dioica* (1D vs. 2D vs. 3D). (**C**-**D**) Venn diagrams showing the differentially abundant metabolites across the three regions for (**C**) *U. hyperborea* and (**D**) *U. dioica.*

Partial Least Squares Discriminant Analysis (PLS-DA), a supervised method incorporating group labels, was used to better distinguish metabolic differences between sample groups. Compared to PCA, PLS-DA offers stronger discriminative power, especially when intergroup differences are subtle. PLS-DA analysis of the distribution of the relative abundances of metabolites indicated clear separation between groups, with no significant within-group separation, confirming representativeness (Fig. 5A and B).

In the *U. dioica* group(1 H vs. 2 H vs. 3 H), the standard for screening DMs was *p* < 0.05. The screening criteria for the *U. dioica* group (1D vs. 2D vs. 3D) are the same as above. In the *U. hyperborea* group, 1,121 DMs were identified; in the *U. dioica* group, 727 DMs were identified. Venn analysis revealed 95 and 21 metabolites whose abundances differed across all three of the sites for *U. hyperborea* and *U. dioica*, respectively (Fig. 5C, D).

### Trend of DMs with altitude

To investigate the potential relationship between altitude and metabolite profiles, K-Means clustering analysis was applied. This method partitions the dataset into distinct clusters, where samples within the same cluster exhibit greater similarity, while those in different clusters show greater dissimilarity.

**Fig. 6:**
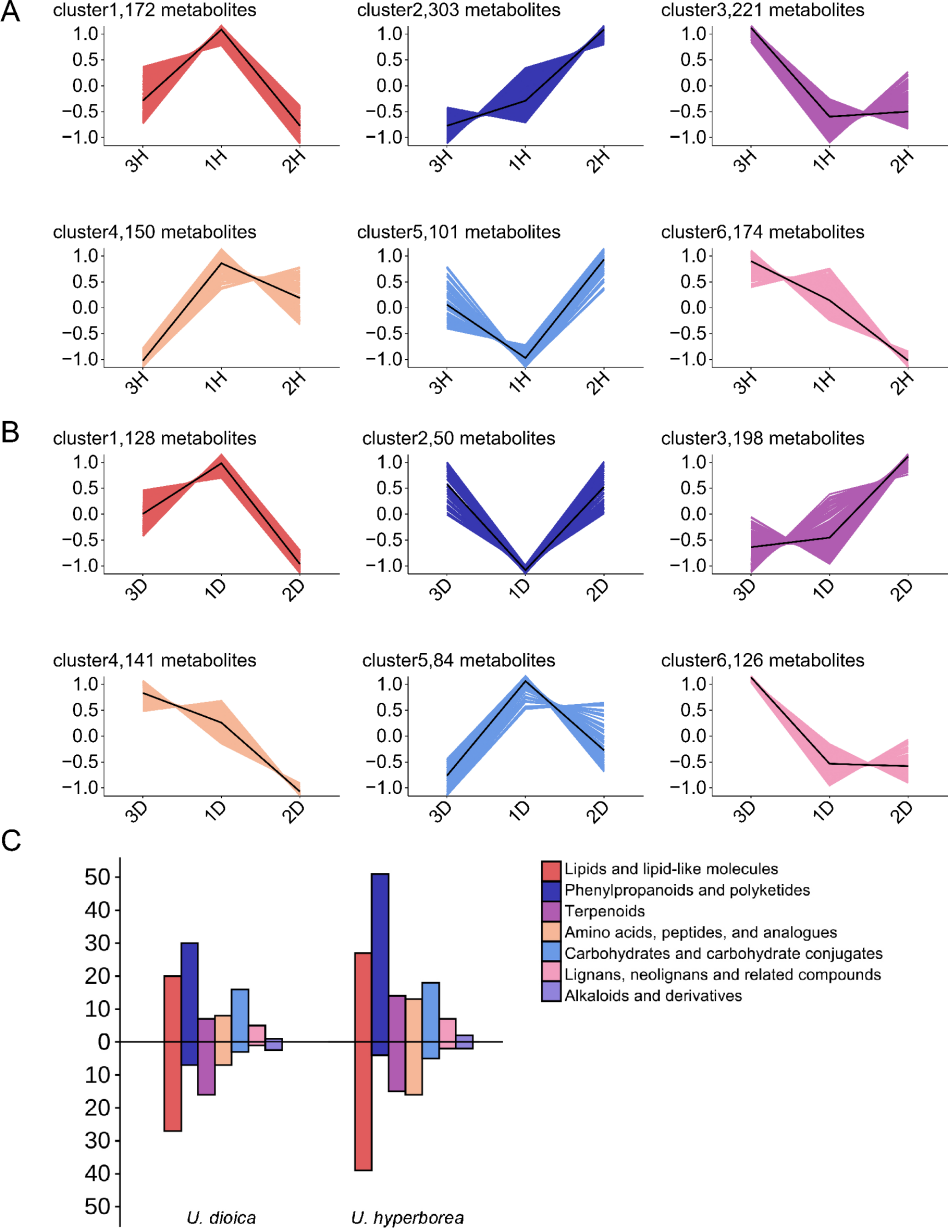
Metabolite differences among clusters of each species across three sites. (**A**-**B**) K-Means clustering analysis of metabolites in (**A**) *Urtica hyperborea* and (**B**) *U. dioica*. (**C**) Metabolite categories in the two species significantly correlated with altitude. Metabolites above the axis exhibit a positive correlation with altitude, while those below the axis show a negative correlation

K-Means clustering analysis categorized the DMs of the *U. hyperborea* group into six distinct clusters based on their patterns of occurrence (Fig. 6A). Cluster 2 included 303 metabolites, which generally increased in abundance as altitude increased. In Cluster 2, the main metabolites included 51 phenylpropanoids and polyketides, 27 lipids and lipid-like molecules, and 18 carbohydrates and carbohydrate conjugates (Fig. 6C). In contrast, Cluster 6 (174 metabolites) displayed a decreasing trend, dominated by 39 lipids and lipid-like molecules, 16 amino acids, peptides, and analogues, and 16 terpenoids (Fig. 6C).

For *U. dioica*, Cluster 3 (198 metabolites) increased significantly with altitude, primarily including 30 phenylpropanoids and polyketides, 20 lipids and lipid-like molecules, and 16 carbohydrates and carbohydrate conjugates. Conversely, Cluster 4 (141 metabolites) exhibited an decreasing trend, with key metabolites being 27 lipids and lipid-like molecules, 17 terpenoids, 7 amino acids, peptides, and analogues, and 7 phenylpropanoids and polyketides (Fig. 6C).

### KEGG annotation and metabolic pathway analysis of DMs at different altitudes

**Fig. 7:**
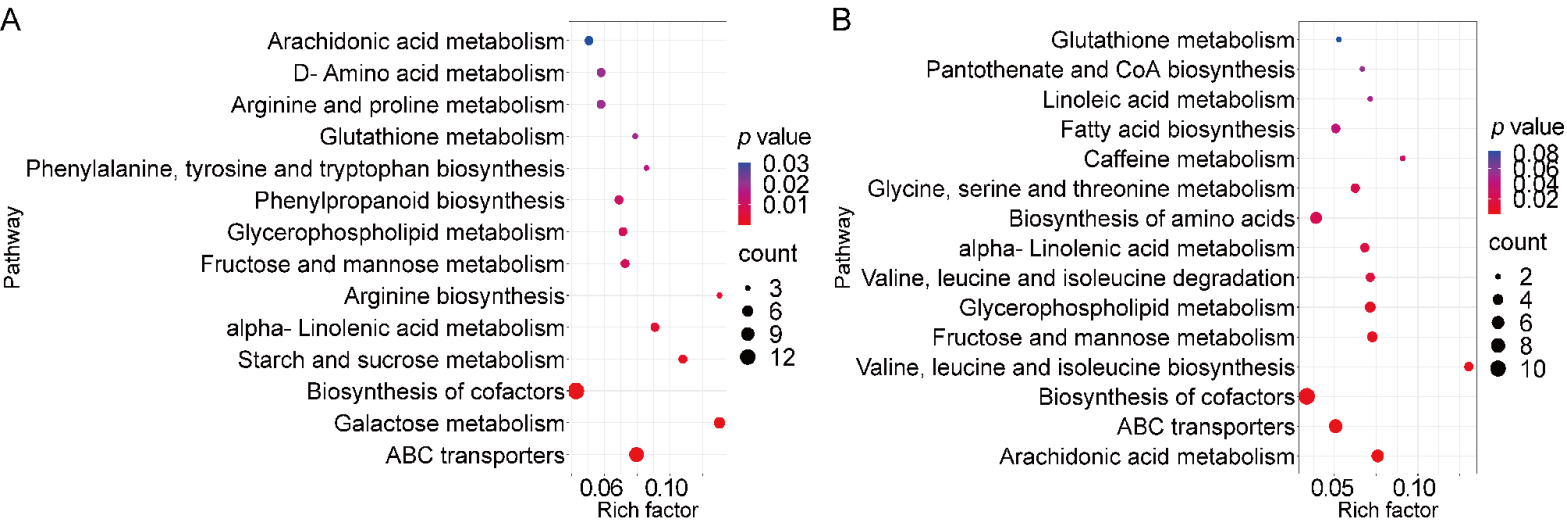
KEGG Pathway Enrichment for metabolites of each species across three sites. Pathway enrichment analysis for *Urtica hyperborea* (**A**) and *U. dioica* (**B**)

After performing KEGG enrichment analysis on the 1121 DMs of the *U. hyperborea* group (1 H, 2 H, 3 H), we obtained 23 significant enrichment pathways with *p* < 0.05 and selected the top 15 pathways to plot by ranking the *p*-values from low to high, as shown in Fig. 7A. For *U. hyperborea*, the key metabolic pathways included D-amino acid metabolism, lysine biosynthesis, and nucleotide metabolism. Similarly, KEGG enrichment analysis was conducted on the 727 DMs of the *U. dioica* group (1D, 2D, 3D). The key metabolic pathways were primarily focused on tyrosine metabolism, 2-Oxocarboxylic acid metabolism, and glyoxylate and dicarboxylate metabolism (Fig. 7B).

## Discussion

### Metabolite differences between species in the same site

Significant metabolic differences were observed between *U. hyperborea* and *U. dioica*, across three different sites on the Tibetan Plateau. These differences indicate that the two species deal with environmental conditions associated with high-altitude in different ways.

In the comparison of the three locations, *U. hyperborea* presented significantly higher levels of phenylpropanoids and polyketides, lipids and lipid-like molecules, and carbohydrates and carbohydrate conjugates compared to *U. dioica*. Only at Site 2 (5007 m) did the metabolic differences between the two species appear relatively small, with *U. dioica* exhibiting a specific upregulation of carbohydrates and carbohydrate conjugates. Compounds such as flavonoids, flavonols, and cinnamic acid, which are phenylpropanoids and polyketides, help high-altitude plants resist UV-B radiation and cold stress [15, 35, 36]. These substances have been found to increase with altitude in studies of *Rhodiola* and *Taxus* [37, 38]. In high-altitude environments, plants often present increased lipids and lipid-like molecules, as well as carbohydrates and carbohydrate conjugates [39, 40]. Lipids and lipid-like molecules primarily assist plants in maintaining normal cell membrane function in low-temperature environments, while the increase in carbohydrates and carbohydrate conjugates reflects the heightened energy demands of high-altitude plants [36, 41].

Through KEGG pathway enrichment analysis, we can draw the following conclusions. At Site 1 and Site 3, the DMs of the two species are concentrated in the pathways of flavonoid biosynthesis, flavone and flavonol biosynthesis, and phenylpropanoid biosynthesis. At the highest altitude Site 2, the metabolic differences between *U. dioica* and *U. hyperborea* are minimal, mainly focused on carbon metabolism pathways such as galactose metabolism, glycolysis/gluconeogenesis, starch and sucrose metabolism, and fructose and mannose metabolism.

Combined with the results of DMs, the content of phenylpropanoids and polyketides in *U. hyperborea* is higher than in *U. dioica*, leading to the enrichment of three related pathways in the KEGG analysis. The enhancement of flavonoid biosynthesis, flavone and flavonol biosynthesis, and phenylpropanoid biosynthesis pathways can improve the antioxidant capacity of plants, helping them resist damage caused by strong ultraviolet radiation in high-altitude areas [42–44]. Phenylpropanoid compounds can also increase the antioxidant capacity of plants, assisting them in overcoming damage induced by low temperatures and oxygen deficiency [16, 45, 46]. Plants in high-altitude environments further adapt by adjusting their carbon metabolism pathways, thereby improving both their antioxidant capacity and energy utilization efficiency [47]. Under drought stress, chickpeas have been shown to exhibit increased levels of metabolites related to glycolysis/gluconeogenesis and galactose metabolism, helping plants maintain energy supply [48]. Starch and sucrose metabolism are associated with plants’ tolerance to, and sensitivity to, abiotic stress [49]. This study is consistent with previous research, enriching four carbon metabolism pathways.

### Metabolite differences in the same species at different altitudes

In the comparison of samples from three different altitudes, the overall metabolic changes in the *U. hyperborea* group (1 H, 2 H, 3 H) and the *U. dioica* group (1D, 2D, 3D) were similar (Fig. 7C), although most of the substances that increase with altitude were more abundant in *U. hyperborea*. The main upregulated compounds include phenylpropanoids and polyketides, lipids and lipid-like molecules, and carbohydrates and carbohydrate conjugates. In contrast, the primary downregulated compounds are lipids and lipid-like molecules, amino acids, peptides, and analogues, and terpenoids.

In most high-altitude plant studies, amino acids, particularly proline, valine, and arginine, generally increase with altitude [24, 37, 50]. However, a few studies report that certain amino acids, such as hydroxyproline, 5-hydroxytryptophan, and carbamyl-aspartate, show a negative correlation with altitude [51, 52]. This may indicate that the decrease in these amino acids contributes to the synthesis of proteins that repair damaged or misfolded structures. In our study, no significant altitudinal trend was observed for amino acids, peptides, and analogues in either *U. hyperborea* or *U. dioica*. This could be due to the sampling points starting as low as 4465 m, with the responses of amino acids, peptides, and analogues to the environment nearing saturation, thus leading to no significant changes in these substances with further increases in altitude.

The increase in phenylpropanoids and polyketides, such as flavonoids, cinnamic acid, and coumarins, helps plants resist strong UV-B radiation in high-altitude areas [53, 54]. Additionally, cinnamic acid and coumarin compounds can regulate gene expression related to oxidative stress, similar to resveratrol, a polyphenolic compound [55]. In both *U. hyperborea* and *U. dioica*, phenylpropanoids and polyketides increase with altitude, which is consistent with previous research.

Carbohydrates and carbohydrate conjugates are considered positive regulators of plant adaptation to various environmental stresses [56, 57]. The increase in photosynthetic rates along altitudinal gradients also leads to higher sugar levels. For instance, *Potentilla saundersiana* has been reported to show increased sugar levels along altitudinal gradients [58]. In the present study, carbohydrates and carbohydrate conjugates in both species exhibited a significant trend of enrichment at high altitudes. This may reflect the plants’ increased energy requirements to adjust their growth cycles and biosynthetic pathways in response to the stresses of high-altitude environments [52].

At high altitudes, plants achieve antibacterial and insect-resistant effects through unsaturated fatty acids, while also improving stress tolerance by enhancing intracellular osmotic regulation through fatty acid derivatives [59, 60]. Both species presented more than twenty lipids and lipid-like molecules positively correlated with altitude. However, in *U. hyperborea*, these were mainly fatty acyls, while in *U. dioica*, they were primarily glycerophospholipids. Fatty acyls are primarily involved in energy storage and participate in plant signalling, whereas glycerophospholipids form the basic structure of cell membranes and can increase membrane fluidity, thus maintaining normal function under low-temperature stress [61]. The differences in these substances further suggest that the adaptation mechanisms of the two species to high-altitude environments differ.

In the study of plant metabolism, the metabolic differences between different species across various geographical regions constitute a significant area of research. In the *U. hyperborea* group (1 H, 2 H, 3 H) and the *U. dioica* group (1D, 2D, 3D), commonly enriched metabolic pathways were observed, including fructose and mannose metabolism, α-linolenic acid metabolism, glycerophospholipid metabolism, and arachidonic acid metabolism. High-altitude environments, such as the Tibetan Plateau, are characterized by low annual average temperatures and significant diurnal temperature fluctuations, which often subject plants to cold stress. This stress can impact the osmotic pressure of plant cells [62]. Fructose and mannose metabolism help plants regulate osmotic pressure and maintain normal cellular functions under such conditions [63]. α-Linolenic acid and arachidonic acid, as unsaturated fatty acids, play crucial roles in the formation of plant cell membranes and in signal transduction pathways [64]. Metabolomic analysis of the roots of winter rapeseed under low-temperature stress revealed significant changes in glycerophospholipid metabolism, particularly an increase in phosphatidylcholine content. This is due to the close association between glycerophospholipid metabolism and the synthesis and repair of cell membranes [65].

In the comparison of the three groups of samples from different altitudes, the metabolic pathways enriched only in the *U. hyperborea* group include starch and sucrose metabolism, galactose metabolism, and phenylpropanoid biosynthesis. The enrichment of starch and sucrose metabolism may be associated with the plant’s adaptation to energy storage and utilization in the high-altitude environment [51, 66]. The enrichment of galactose metabolism may be related to the synthesis and modification of plant cell walls, which is crucial for the growth and development of plants in high-altitude conditions [47, 67]. The enrichment of phenylpropanoid biosynthesis may be linked to the plant’s stress resistance and antioxidant defense mechanisms [36].

The metabolically enriched pathways specific to *U. dioica* included pantothenate and coenzyme A biosynthesis, as well as glutathione metabolism. Pantothenate and coenzyme A are crucial members of the vitamin B complex, participating in numerous enzymatic reactions, including those related to the metabolism of sugars, fats, and proteins [68]. Glutathione, an essential antioxidant, plays a role in the plant’s defense against oxidative stress [69, 70]. This suggests *U. dioica* may adapt to its growth environment and enhance its survival capabilities by regulating these metabolic pathways.

The differential enrichment of metabolites between the two species in different geographical regions reflects their adaptive strategies in specific environments. They can optimize energy utilization, strengthen defense mechanisms, and improve survival by modulating particular metabolic pathways, such as fructose and mannose metabolism, and α-linolenic acid metabolism [39, 71]. Additionally, the enrichment of these metabolic pathways reveals the evolutionary diversity and adaptability of plant secondary metabolic pathways.

## Conclusion

In this study, a total of 2906 annotated metabolites were detected through comparative metabolomic analyses of *U. hyperborea* and *U. dioica* across three sites. In the comparison of metabolite differences between sympatric species, 355 differential metabolites (DMs) were identified at Site 1; 76 DMs at Site 2; and 373 DMs at Site 3. At Sites 1 and 3, these DMs were primarily concentrated in the pathways of flavonoid biosynthesis, flavone and flavonol biosynthesis, and phenylpropanoid biosynthesis. In contrast, at Site 2, the highest altitude, the DMs were predominantly enriched in carbon metabolism pathways. Furthermore, comparing metabolic changes of the same species across different altitudes revealed commonly enriched pathways, including fructose and mannose metabolism, α-linolenic acid metabolism, glycerophospholipid metabolism, and arachidonic acid metabolism. Thus, our study reveals that the high-altitude adaptation mechanisms of sympatric species are not identical. The adaptation mechanisms of the same species at different locations are largely similar, with slight variations. These findings provide metabolic insights that contribute to our understanding of plant adaptation mechanisms to high-altitude environments. Moreover, by examining the unique adaptive strategies of closely related species, our findings highlight the metabolic diversity that enables plants to thrive under extreme environmental conditions.

## Electronic supplementary material

Below is the link to the electronic supplementary material.

Supplementary Material 1

Supplementary Material 2

Supplementary Material 3

Supplementary Material 4

Supplementary Material 5

## Data Availability

All data presented in this research are available in the article and supplementary materials.

## References

[1] Yao TD, Chen FH, Cui P, Ma YM, Xu BQ, Zhu LP, Zhang F, Wang WC, Ai LK, Yang XX. From Tibetan plateau to third pole and Pan-Third pole. BCAS. 2017;32:924–31.

[2] Liu J, Milne RI, Zhu GF, Spicer RA, Wambulwa MC, Wu ZY, et al. Name and scale matter: clarifying the geography of Tibetan Plateau and adjacent mountain regions. Global Planet Change. 2022;215:103893.

[3] Zhang C, An YM, Yun JÄSCHKE, Wang LL, Zhou ZL, Wang LP, Yang YP, Duan YW. Processes on reproductive ecology of plant species in the Qinghai-XizangPlateau and adjacent highlands. Chin J Plant Ecol. 2020;44:1–21.

[4] Song B, Zhang ZQ, Stöcklin J, Yang Y, Niu Y, Chen JG, et al. Multifunctional bracts enhance plant fitness during flowering and seed development in *Rheum nobile* (Polygonaceae), a giant herb endemic to the high Himalayas. Oecologia. 2013;172:359–70. 23124332 10.1007/s00442-012-2518-2

[5] Peng DL, Niu Y, Song B, Chen JG, Li ZM, Yang Y, et al. Woolly and overlapping leaves dampen temperature fluctuations in reproductive organ of an alpine Himalayan forb. J Plant Ecol. 2015;8:159–65.

[6] Myers N, Mittermeierr A, Mittermeierc G, Dafonsecaga B, Kent J. Biodiversity hotspots for conservation priorities. Nature. 2000;403:853–8. 10706275 10.1038/35002501

[7] Chen CJ, Lin Q, Friis I, Wilmot-Dear CM, Monro AK. Urticaceae. In: Flora of China (eds. Wu ZY, Raven PH). Bejing: Science Press, Bejing & Missouri Botanical Garden Press; 2003. pp. 76–189.

[8] Wu Z-Y, Monro AK, Milne RI, Wang H, Yi T-S, Liu J, et al. Molecular phylogeny of the nettle family (Urticaceae) inferred from multiple loci of three genomes and extensive generic sampling. Mol Phylogenet Evol. 2013;69:814–27. 23850510 10.1016/j.ympev.2013.06.022

[9] Ogoma CA, Liu J, Stull GW, Wambulwa MC, Oyebanji O, Milne RI et al. Deep insights into the plastome evolution and phylogenetic relationships of the tribe urticeae (Family Urticaceae). Front. Plant Sci. 2022;13. 10.3389/fpls.2022.870949 PMC9164014 35668809

[10] Taylor K. Biological flora of the British Isles: *Urtica dioica* L. J Ecol. 2009;97:1436–58.

[11] Uemura M, Raymond A, Joseph, Steponkus PL. Cold acclimation of *Arabidopsis thaliana* (effect on plasmamembrane lipid composition and freeze-induced lesions. Plant Physiol. 1995;109(1):15–30. 12228580 10.1104/pp.109.1.15 PMC157560

[12] Walker DJ, Romero P, Correal E. Cold tolerance, water relations and accumulation of osmolytes in *Bituminaria bituminosa*. Biol Plant. 2010;54:293–8.

[13] Klotke J, Kopka J, Gatzke N, Heyer AG. Impact of soluble sugar concentrations on the acquisition of freezing tolerance in accessions of *Arabidopsis thaliana* with contrasting cold adaptation– evidence for a role of raffinose in cold acclimation. Plant Cell Environ. 2004;27:1395–404.

[14] Xie H, Wang Q, Zhang P, Zhang X, Huang T, Guo Y, et al. Transcriptomic and metabolomic analysis of the response of Quinoa seedlings to low temperatures. Biomolecules. 2022;12(7):977–94. 35883533 10.3390/biom12070977 PMC9312504

[15] Santin M, Ranieri A, Hauser M-T, Miras-Moreno B, Rocchetti G, Lucini L, et al. The outer influences the inner: postharvest UV-B irradiation modulates Peach flesh metabolome although shielded by the skin. Food Chem. 2021;338:127782. 32798826 10.1016/j.foodchem.2020.127782

[16] Yan Y, Stoddard FL, Neugart S, Sadras VO, Lindfors A, Morales LO, et al. Responses of flavonoid profile and associated gene expression to solar blue and UV radiation in two accessions of *Vicia faba* L. from contrasting UV environments. Photoch Photobio Sci. 2019;18:434–47. 10.1039/c8pp00567b 30629071

[17] Zhang X, Kuang T, Dong W, Qian Z, Zhang H, Landis JB, et al. Genomic convergence underlying high-altitude adaptation in alpine plants. J Integr Plant Biol. 2023;65:1620–35. 36960823 10.1111/jipb.13485

[18] Ledig FT, Korbobo DR. Adaptation of sugar maple populations along altitudinal gradients: photosynthesis, respiration, and specific weight. Amer J Bot. 1983;70:256–65.

[19] Zhu X, Zhang M, Wang B, Song X, Wang X, Wei X. Non-targeted metabolomics analysis of metabolite changes in two Quinoa genotypes under drought stress. BMC Plant Biol. 2023;23:503–21. 37858063 10.1186/s12870-023-04467-6 PMC10588040

[20] Singh V, Gupta K, Singh S, Jain M, Garg R. Unravelling the molecular mechanism underlying drought stress response in Chickpea via integrated multi-omics analysis. Front Plant Sci. 2023;14:1–19. 10.3389/fpls.2023.1156606 PMC10242046 37287713

[21] Cao P, Yang J, Xia L, Zhang Z, Wu Z, Hao Y, et al. Two gene clusters and their positive regulator SlMYB13 that have undergone domestication-associated negative selection control phenolamide accumulation and drought tolerance in tomato. Mol Plant. 2024;17(4):579–97. 38327054 10.1016/j.molp.2024.02.003

[22] Jia XH, Wu FM, Lu AJ, Tan DP, Zhang QR, He YQ et al. Widely targeted metabolomics analysis of *Dendrobium officinale* at different altitudes. Chem Biodivers 2023, 20, e202201082. 10.1002/cbdv.202201082 36891987

[23] Lu Q, Li R, Liao J, et al. Integrative analysis of the steroidal alkaloids distribution and biosynthesis of bulbs *Fritillariae cirrhosae* through metabolome and transcriptome analyses. BMC Genomics. 2022. 10.1186/s12864-022-08724-0. 35836113 10.1186/s12864-022-08724-0 PMC9284883

[24] Lei L, Yuan X, Fu K, Chen Y, Lu Y, Shou N, et al. Pseudotargeted metabolomics revealed the adaptive mechanism of *Draba oreades* Schrenk at high altitude. Front Plant Sci. 2022;13:1052640. 10.3389/fpls.2022.1052640 36570906 10.3389/fpls.2022.1052640 PMC9784223

[25] Wang Z, Li P, Jia Y, et al. Integrated metabolomic and transcriptomic analysis of triterpenoid accumulation in the roots of *Codonopsis pilosula* Var. *Modesta* (Nannf.) L.T.Shen at different altitudes. Phytochem Anal. 2025;36(2):358–68. 10.1002/pca.3362. 38764207 10.1002/pca.3362

[26] Wang JY, Yang ML, Huang XQ, et al. Response of leaf blade in *Zanthoxylum planispinum* Var. Dintanensis to differences altitudes through integrated metabolomics and transcriptomics analysis. South Afr J Bot. 2023;162:41–51.

[27] Fraga CG, Clowers BH, Moore RJ, Zink EM. Signature-Discovery approach for sample matching of a Nerve-Agent precursor using liquid Chromatography– Mass spectrometry, XCMS, and chemometrics. Anal Chem. 2010;82:4165–73. 20405949 10.1021/ac1003568

[28] Zhou Z, Luo M, Zhang H, Yin Y, Cai Y, Zhu ZJ. Metabolite annotation from knowns to unknowns through knowledge-guided multi-layer metabolic networking. Nat Commun. 2022;13(1):6656. 36333358 10.1038/s41467-022-34537-6 PMC9636193

[29] Sumner LW, Amberg A, Barrett D, et al. Proposed minimum reporting standards for chemical analysis chemical analysis working group (CAWG) metabolomics standards initiative (MSI). Metabolomics. 2007;3(3):211–21. 10.1007/s11306-007-0082-2. 24039616 10.1007/s11306-007-0082-2 PMC3772505

[30] Shirzadifar A, Bajwa S, Mireei SA, Howatt K, Nowatzki J. Weed species discrimination based on SIMCA analysis of plant canopy spectral data. Biosyst Eng. 2018;171:143–54.

[31] Chen H, Boutros PC. VennDiagram: a package for the generation of highly-customizable Venn and Euler diagrams in R. BMC Bioinform. 2011;12:35–41. 10.1186/1471-2105-12-35 PMC3041657 21269502

[32] Ogata H, Goto S, Sato K, Fujibuchi W, Bono H. KEGG: Kyoto encyclopedia of genes and genomes. Nucleic Acids Res. 1999;27:29–34. 9847135 10.1093/nar/27.1.29 PMC148090

[33] Trygg J, Wold S. Orthogonal projections to latent structures (O-PLS). J Chemom. 2002;16:119–28.

[34] TÃ¶pfer N, Kleessen S, Nikoloski Z. Integration of metabolomics data into metabolic networks. Front Plant Sci. 2015;6:1–13. 25741348 10.3389/fpls.2015.00049 PMC4330704

[35] Liu XW, Wang YH, Shen SK. Transcriptomic and metabolomic analyses reveal the altitude adaptability and evolution of different-colored flowers in alpine *Rhododendron* species. Tree Physiol. 2021;42(5):1100–13. 10.1093/treephys/tpab160 34850945

[36] Nataraj N, Hussain M, Ibrahim M, Hausmann AE, Rao S, Kaur S, et al. Effect of altitude on volatile organic and phenolic compounds of *Artemisia brevifolia* wall ex Dc. from the Western Himalayas. Front Ecol Evol. 2022;10:1–10.

[37] Dong X, Guo Y, Xiong C, Sun L. Evaluation of two major *Rhodiola* species and the systemic changing characteristics of metabolites of *Rhodiola crenulata* in different altitudes by chemical methods combined with UPLC-QqQ-MS-Based metabolomics. Molecules. 2020;25:4062. 32899531 10.3390/molecules25184062 PMC7570721

[38] Yu C, Luo X, Zhan X, Hao J, Zhang L, Song Y-BL, et al. Comparative metabolomics reveals the metabolic variations between two endangered *Taxus* species (*T. fuana* and *T. yunnanensis*) in the Himalayas. BMC Plant Biol. 2018;18:197–208. 30223770 10.1186/s12870-018-1412-4 PMC6142684

[39] Weng JK, Philippe RN, Noel JP. The rise of chemodiversity in plants. Science. 2012;336(6089):1667–70. 22745420 10.1126/science.1217411

[40] Paine AK, Mitra S, Bera R, Paul I, Sarkar MP. Metabolic insights into high-altitude adaptation of Himalayan ‘horsetails’ [*Equisetum diffusum* D. Don] with special reference to the fatty acid dynamicity. S Afr J Bot. 2024;171:267–76.

[41] Pichersky E, Lewinsohn E. Convergent evolution in plant specialized metabolism. Annu Rev Plant Biol. 2011;62:549–66. 21275647 10.1146/annurev-arplant-042110-103814

[42] Liu ZH, Liu YX, Pu ZE, Wang JR, Zheng YL, Li YH, et al. Regulation, evolution, and functionality of flavonoids in cereal crops. Biotechnol Lett. 2013;35:1765–80. 23881316 10.1007/s10529-013-1277-4

[43] Grisebach H. Compative biosynthetic pathways in higher plants. Pure Appl Chem. 1973;34(3–4):487–514.

[44] Bornman JF, Reuber S, Cen YP, Weissenböck G. Ultraviolet radiation as a stress factor and the role of protective pigments. Cambridge University Press; 1997. 10.1017/cbo9780511752346.010.

[45] Li P, Li QF, Huang YY, Huang RD. The advance of plant flavonoids of anti-UV-B radiation research. Chin J Ecol. 2001;20(6):36–40.

[46] Zeng XQ, Yuan HJ, Dong XK, Peng M, Jing XY, Xu QJ, et al. Genome-wide dissection of co-selected UV-B responsive pathways in the UV-B adaptation of Qingke. Mol. Plant. 2020;13:112–27. 10.1016/j.molp.2019.10.009 31669581

[47] Tess SD, Nelle V, Madera M, P.Vogel J, Dahlberg J,Hutmacher R, et al. Cell wall compositions of Sorghum bicolor leaves and roots remain relatively constant under drought conditions[J]. Frontiers in Plant Science. 2021, 12. 10.3389/fpls.2021.747225 10.3389/fpls.2021.747225 PMC8632824 34868130

[48] Sharma KD, Patil G, Kiran A. Characterization and differential expression of sucrose and starch metabolism genes in contrasting Chickpea (*Cicer arietinum* L.) genotypes under low temperature. J Genet. 2021;100(71):1–14. 34608872

[49] Huang MZ, Zhang X, Yan WJ, Liu JJ, Wang H. Metabolomics reveals potential plateau adaptability by regulating infammatory response and oxidative stress-related metabolism and energy metabolism pathways in Yak. J Anim Sci Technol. 2022;64(1):97–109. 35174345 10.5187/jast.2021.e129 PMC8819316

[50] Kumar V, Kumar P, Bhargava B, Sharma R, Irfan M, Chandora R. Transcriptomic and metabolomic reprogramming to explore the high-altitude adaptation of medicinal plants: A review. J Plant Growth Regul. 2023;42:7315–29.

[51] Zhao Y, Xu F, Liu J, Guan F, Quan H, Meng F. The adaptation strategies of *Herpetospermum pedunculosum* (Ser.) Baill at altitude gradient of the Tibetan Plateau by physiological and metabolomic methods. BMC Genomics. 2019;20:451–65. 31159723 10.1186/s12864-019-5778-y PMC6547600

[52] Shi JS, Gu R, Chen SL, Zhang C, Guo ZW. The effect of altitude on the protein nutritional value of *Phyllostachys* prominens bamboo shoots. Acta Agriculturae Universitatis Jiangxiensis. 2019;2:308–15.

[53] Lattanzio V, Cardinali A, Linsalata V, Plant Phenolics: A Biochemical and Physiological Perspective. In Recent Advances in Polyphenol Research (eds S. Quideau, V. Cheynier, P. Sarni-Manchado and S. Quideau). 2012; 10.1002/9781118299753.ch1

[54] Ziska LH, Teramura AH, Sullivan JH. Physiological sensitivity of plants along an elevational gradient to UV-B radiation. Am J Bot. 1992;79:863–71.

[55] Groote DD, Belleghem KV, Devière J, Brussel WV, Mukaneza A, Amininejad L. Effect of the intake of resveratrol, resveratrol phosphate, and catechin-rich grape seed extract on markers of oxidative stress and gene expression in adult obese subjects. Ann Nutr Metab. 2012;61:15–24. 22776850 10.1159/000338634

[56] Rizhsky L, Liang HJ, Shuman J, et al. When defense pathways collide. The response of *Arabidopsis* to a combination of drought and heat stress. Plant Physiol. 2004;134:1683–96. 15047901 10.1104/pp.103.033431 PMC419842

[57] Wulff C, Gatzke N, Kopka J, Orellana A, Hoefgen R, Fisahn J, et al. Photosynthesis and metabolism interact during acclimation of *Arabidopsis thaliana* to high irradiance and sulphur depletion. Plant Cell Environ. 2010;33(11):1974–88. 20573050 10.1111/j.1365-3040.2010.02199.x

[58] Tang X, Li J, Liu L, Jing H, Zuo W, Zeng Y. Transcriptome analysis provides insights into *Potentilla bifurca* adaptation to high altitude. Life. 2022;12:1337–54. 36143374 10.3390/life12091337 PMC9503701

[59] Cui G, Wei X, Degen AA, Wei X, Zhou J, Ding L, et al. Trolox-equivalent antioxidant capacity and composition of five alpine plant species growing at different elevations on the Qinghai–Tibetan Plateau. Plant Ecol Divers. 2016;9:387–96.

[60] Liu FJ, Yu S, Liu G. Mechanism and research progress of plant lipid regulation under biotic and abiotic stresses. Chin J Oil Crop Sci. 2023;45(5):1062–72.

[61] Zheng G, Tian B, Zhang F, Tao F, Li W. Plant adaptation to frequent alterations between high and low temperatures: remodelling of membrane lipids and maintenance of unsaturation levels. Plant Cell Environ. 2011;34:1431–42. 21486310 10.1111/j.1365-3040.2011.02341.x PMC3980542

[62] Calzadilla PI, Signorelli S, Maiale SJ. Photosynthetic responses mediate the adaptation of two *Lotus japonicus* ecotypes to low temperature. Plant Sci. 2016;250:59–68. 27457984 10.1016/j.plantsci.2016.06.003

[63] Bustamante CA, Monti LL, Gabilondo J, Scossa F, Valentini G, Budde CO et al. Differential metabolic rearrangements after cold storage are correlated with chilling injury resistance ofeach fruits. Front. Plant Sci. 2016;7. 10.3389/fpls.2016.01478 PMC5044465 27746802

[64] Zhu XL, Zhang MJ, Wang BQ, Song XR, Wang X, Wei XH. Non-targeted metabolomics analysis of metabolite changes in two quinoa genotypes under drought stress. BMC Plant Biol. 2023;23:503–21. 37858063 10.1186/s12870-023-04467-6 PMC10588040

[65] Junyan W, Qiaowen P, Fahim AM, Lulu Z, Hui G, Lijun L, et al. Effects of exogenous calcium and calcium inhibitor on physiological characteristics of winter turnip rape (*Brassica rapa*) under low temperature stress. BMC Plant Biol. 2024;24:937. 39385096 10.1186/s12870-024-05556-w PMC11462862

[66] Huang W, Han S, Wang L, Li W. Carbon and nitrogen metabolic regulation in freshwater plant Ottelia alismoides in response to carbon limitation: A metabolite perspective. Front. Plant Sci. 2022;13. 10.3389/fpls.2022.962622 PMC9522611 36186073

[67] Avasthi AS, Kaur N, Sharda S, Ghosal S. An insight into antioxidant and antimicrobial activities of ethnotherapeutically important trans Himalayan medicinal plants: A review. J Pharm Res Int. 2021;33(36A):195–212.

[68] Wang KH, Zhu B, Zhu ZJ. Review of the role of GSH/GSSG in plant abiotic stress response. Acta Hortic Sinica. 2021;48(4):647–60.

[69] Szalai G, Kellős T, Galiba G, Kocsy G. Glutathione as an antioxidant and regulatory molecule in plants under abiotic stress conditions. J Plant Growth Regul. 2009;28:66–80.

[70] D’’Oria A, Jing L, Arkoun M, Pluchon S, Pateyron S. Transcriptomic, metabolomic and ionomic analyses reveal early modulation of leaf mineral content in *Brassica napus* under mild or severe drought. Int Int J Mol Sci. 2022;23(2):781–806. 35054964 10.3390/ijms23020781 PMC8776245

[71] Maeda HA. Evolutionary diversification of primary metabolism and its contribution to plant chemical diversity. Front Plant Sci. 2019;10:1–8. 31354760 10.3389/fpls.2019.00881 PMC6635470

