# Supplementary figures and images for: Comparative metabolomics of two nettle species unveils distinct high-altitude adaptation mechanisms on the Tibetan Plateau
Source: BMC Plant Biol. 2025 May 15;25:640. doi: 10.1186/s12870-025-06666-9 (PMC12079973; doi:10.1186/s12870-025-06666-9)

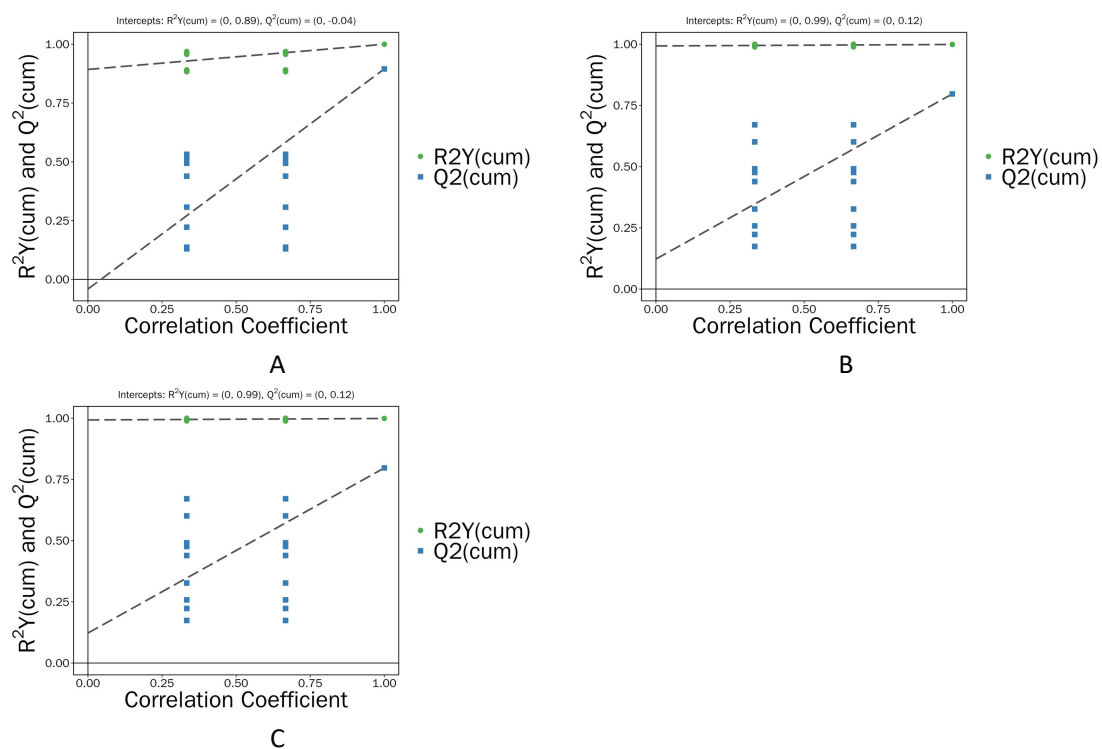

**Fig S3.** OPLS-DA permutation plot. (A) Site 1 (1H vs. 1D), (B) Site 2 (2H vs. 2D), and (C) Site 3 (3H vs. 3D).

Supplement: Supplementary file 3 — Supplementary Material 3 [file 12870_2025_6666_MOESM3_ESM.pdf]

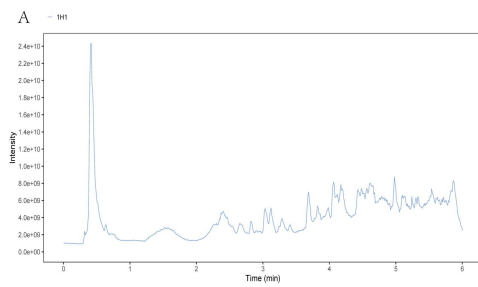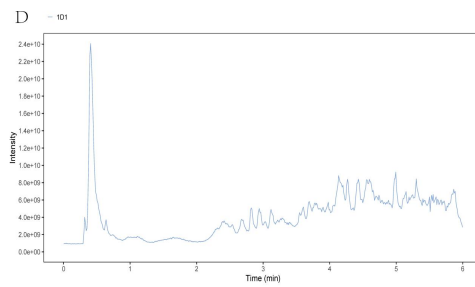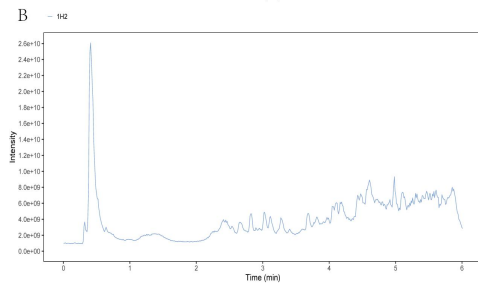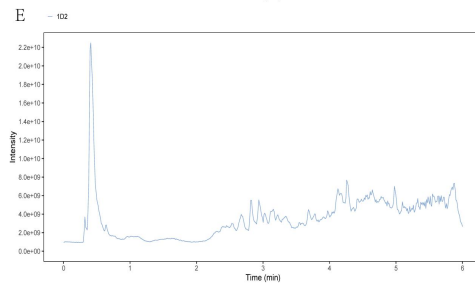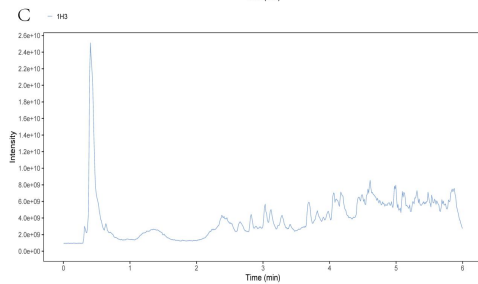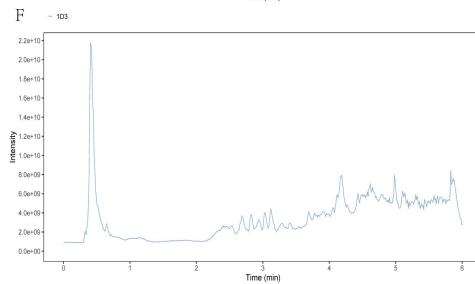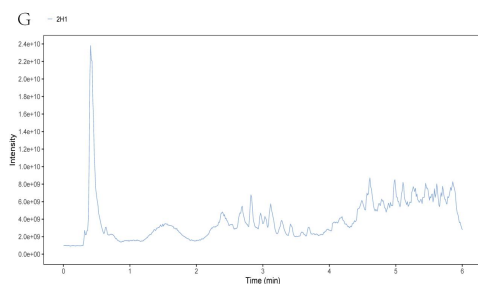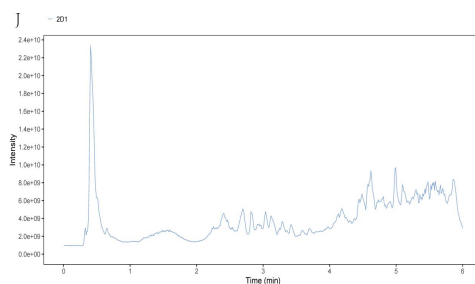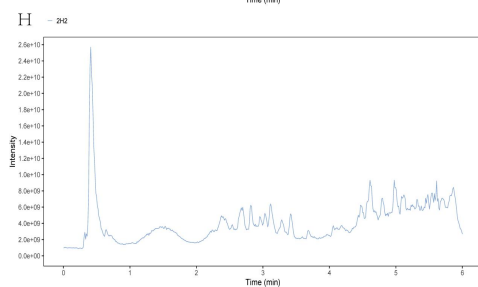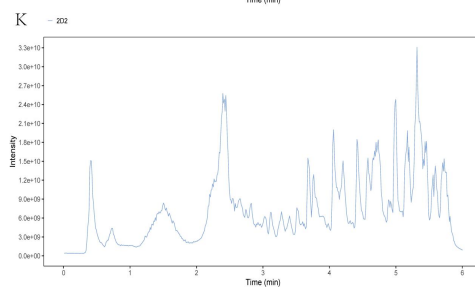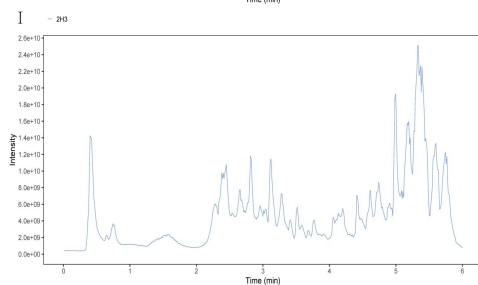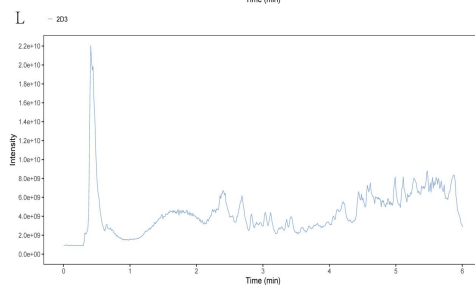

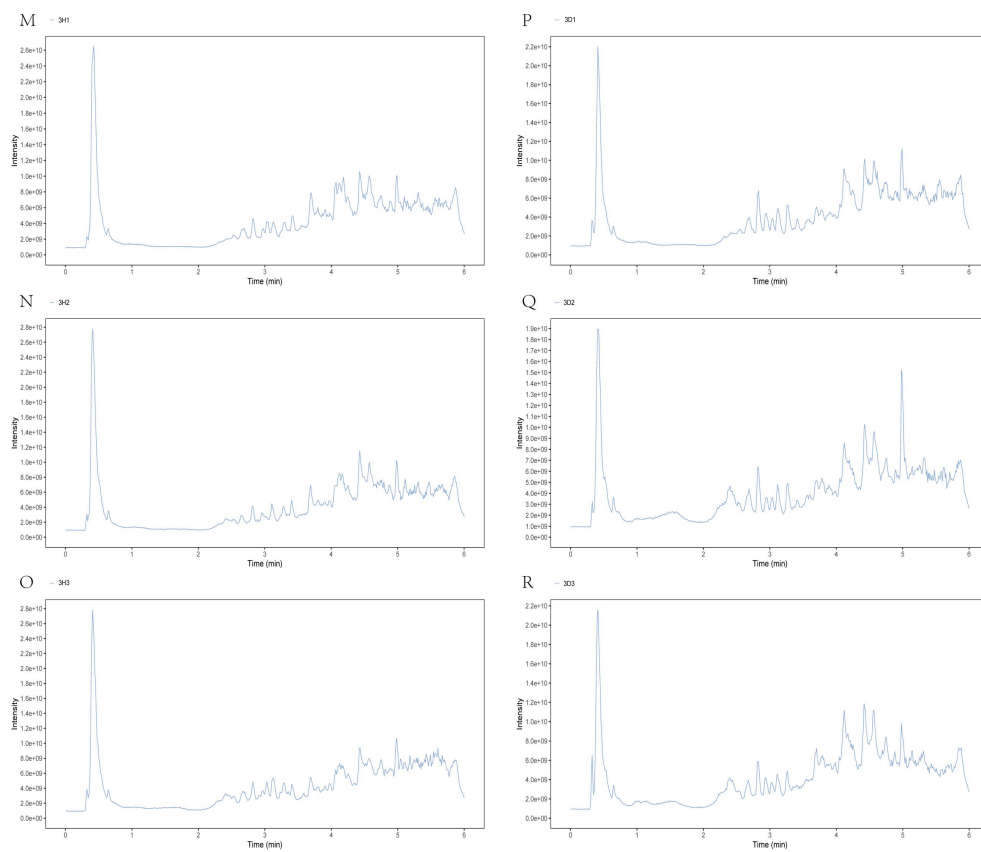

**Fig S1.** Total Ion Chromatogram (TIC) in Positive Ion Mode.

Supplement: Supplementary file 4 — Supplementary Material 4 [file 12870_2025_6666_MOESM4_ESM.pdf]

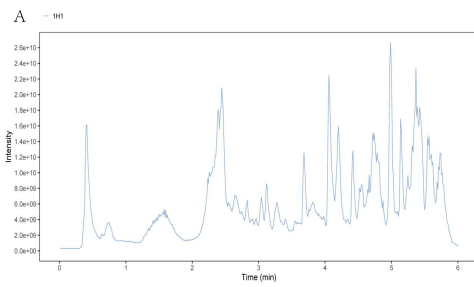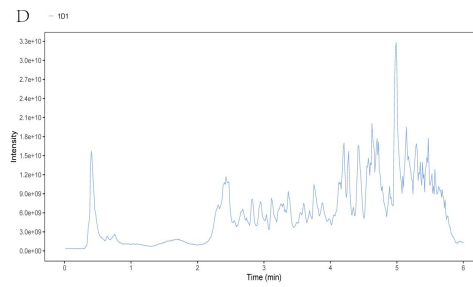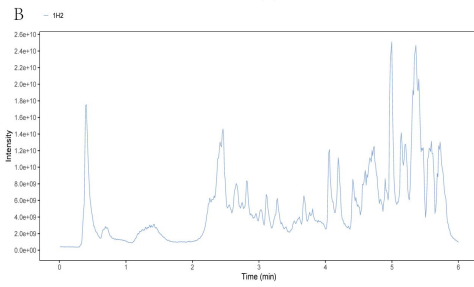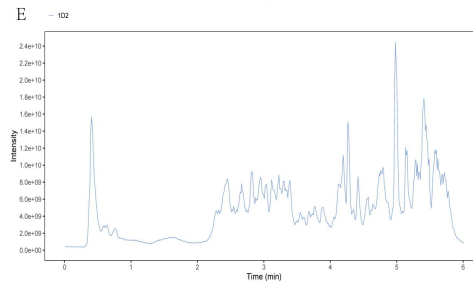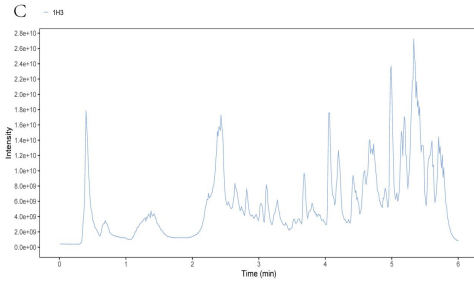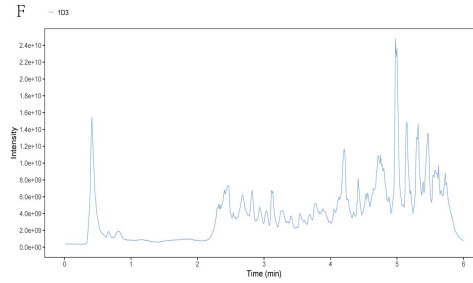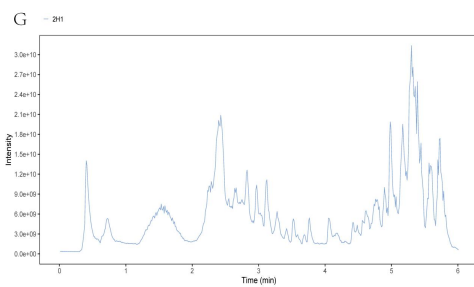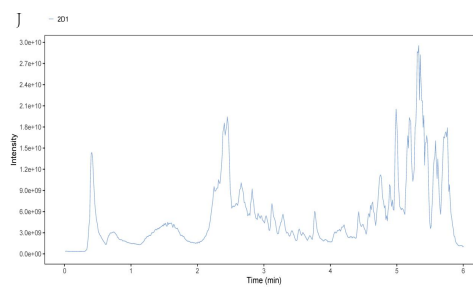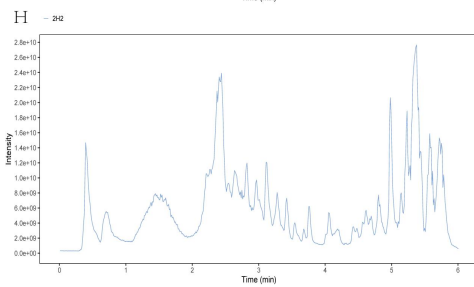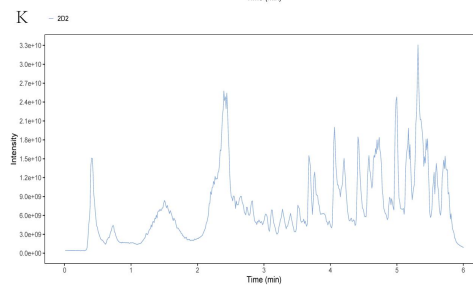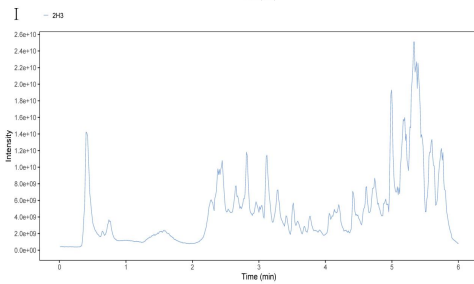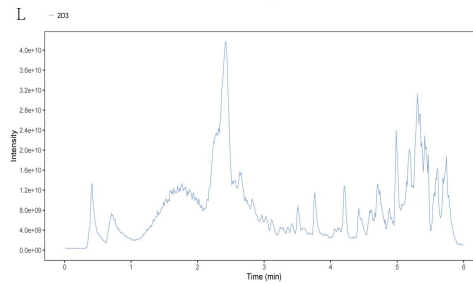

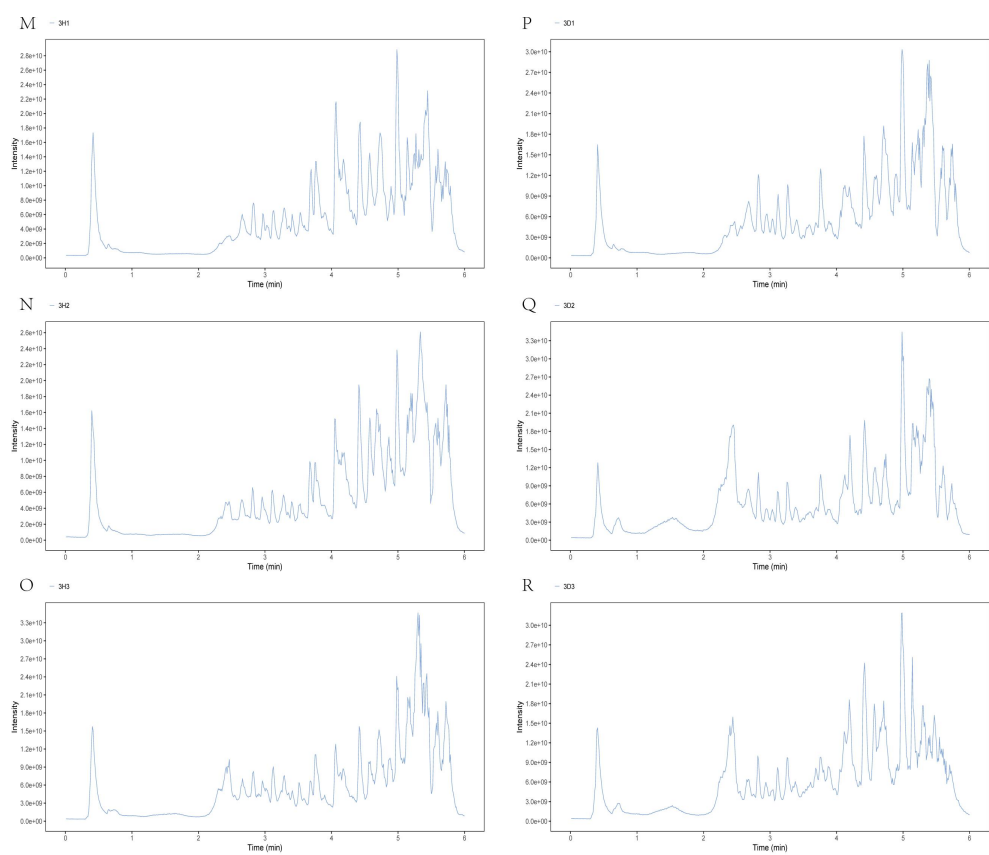

**Fig S2.** Total Ion Chromatogram (TIC) in Negative Ion Mode

Supplement: Supplementary file 5 — Supplementary Material 5 [file 12870_2025_6666_MOESM5_ESM.pdf]
